# Health-related quality of life and influencing factors of patients with paroxysmal nocturnal hemoglobinuria in China

**DOI:** 10.1186/s13023-024-03178-x

**Published:** 2024-05-03

**Authors:** Huaxin Yu, Shengnan Duan, Pei Wang, Rong Fu, Zixuan Lv, Yuchi Yu, Pu Miao, Junwei Shi, Niekun Zhuang, Huiying Hu, Ni Yuan, Sijia Che

**Affiliations:** 1 School of Public Health, Dalian Medical University, Dalian, China; 2 School of Public Health, Fudan University, 130 Dong An Road, Shanghai, China; 3 Tianjin Medical University General Hospital, 154 Anshan Road, Tianjin, China; 4 PNH China, Shenzhen, China; 5 Global Health Research Center, Dalian Medical University, 9 Lvshun South Road, Dalian, Liaoning China

**Keywords:** Paroxysmal nocturnal hemoglobinuria, Health-related quality of life (HRQOL), EQ-5D-5L, Influencing factors, China

## Abstract

**Background:**

Paroxysmal nocturnal hemoglobinuria (PNH) is a rare blood disorder, leading to various complications and impairments in patients’ health-related quality of life (HRQOL). Limited research has been conducted to evaluate the HRQOL of Chinese patients with PNH. Understanding the HRQOL in this specific population is crucial for providing effective healthcare interventions and improving patient’ health outcomes. This study aimed to assess HRQOL of Chinese patients with PNH, and identify key determinants.

**Methods:**

A cross-sectional study was conducted during 2022 to recruit patients with PNH in China. The study population was recruited from PNH China, one of the largest public welfare PNH patient mutual aid organization in China. Data were collected via an online questionnaire including the EQ-5D-5L (5L), and social-demographic and clinical characteristics. Descriptive statistics were employed to summarize the characteristics of the participants and their HRQOL. Multiple linear and logistic regression analyses were adopted to explore key factors affecting HRQOL.

**Results:**

A total of 329 valid questionnaires were collected. The mean (SD) age of the patients was 35.3 (10.0) years, with 52.3% of them being male. The patients reported more problems in Anxiety/Depression (81.5%) and Pain/Discomfort (69.9%) dimensions compared to the other three 5L dimensions. The mean (SD) of 5L health utility score (HUS) and EQ-VAS score were 0.76 (0.21) and 62.61 (19.20), respectively. According to multiple linear regression, initial symptoms (i.e., Anemia [fatigue, tachycardia, shortness of breath, headache] and back pain) and complication of thrombosis were significant influencing factors affecting 5L HUS. Total personal income of the past year, initial symptom of hemoglobinuria and complication of thrombosis were significantly influencing factors of VAS score. Social-demographic and clinical characteristics, such as gender, income, and thrombosis, were also found to be significantly related to certain 5L health problems as well.

**Conclusion:**

Our study manifested the HRQOL of PNH patients in China was markedly compromised, especially in two mental-health related dimensions, and revealed several socio-demographic and clinical factors of their HRQOL. These findings could be used as empirical evidence for enhancing the HRQOL of PNH patients in China.

**Supplementary Information:**

The online version contains supplementary material available at 10.1186/s13023-024-03178-x.

## Background

Paroxysmal nocturnal hemoglobinuria (PNH) is an acquired hematopoietic stem cell disorder, resulting from a somatic mutation in the X-linked gene phosphatidylinositol glycan class A (PIGA) which leads to the expansion of hematopoietic stem cell clones [1, 2]. PNH, an ultra-orphan disease due to its rarity, manifests as a chronic, multi-systemic, and progressive illness posing life-threatening risks, characterized by hemolytic anemia, hemoglobinuria, thrombotic events (TEs), severe infections, smooth muscle dystonia and bone marrow failure (BMF) [1–8]. The global prevalence of PNH is estimated to range between 10 and 20 cases per million [5]. Evidence also suggests that the worldwide incidence of PNH is estimated at 1-1.5 cases per million, with a potentially higher rate in certain regions [2, 9, 10]. For example, epidemiological data show that the disease occurs more frequently in Asia countries (e.g., Japan, Korea and China) than in western countries (e.g., US, UK, Spain) [9–11]. However, there is a lack of nationwide data in China, though one regional study in Mudanjiang region of Heilongjiang Province showed that the standardized incidence was 2.7 per 100,000 population [12].

PNH can affect individuals at any age group, showing no significant preferences towards gender, ethnicity, or geographical region. Nevertheless, it manifests most commonly in young adulthood, with the median diagnosis age in the early- to mid-thirties [5, 7, 11, 12]. Despite optimal supportive care, retrospective analyses report a substantial increase in mortality due to PNH, with 5-year and 10-year mortality rates estimated at 35% and approximately 50%, respectively [1, 5, 6].

PNH has an extensive adverse impact on various health aspects of patients. According to the International PNH Registry, PNH patients frequently experience symptoms of fatigue, headache, dyspnea, hemoglobinuria, abdominal pain, erectile dysfunction and dysphagia [13, 14]. Hence, it is essential to enhance or sustain the patients’ health-related quality of life (HRQOL), a multidimensional health outcome generally consisting of the physical, social and emotional aspects of health perception and functioning [15–17]. HRQOL information on patients with PNH could be valuable in facilitating healthcare, evaluating disease burden, and assessing the effectiveness/cost-effectiveness of health interventions (if utility instruments are adopted) for the patients.

Empirical evidence has suggested that PNH can significantly reduce HRQOL of the patients in western populations [13, 14, 18–22]. Given the disparities in medical systems, cultural background, and patient characteristics between China and western countries, the existing evidence may not be directly applicable to China. Hence, our aim was to comprehensively evaluate HRQOL of Chinese patients with PNH using the EQ-5D-5L (5L), a new version of the widely used EQ-5D, and to explore the influencing factors.

## Methods

### Aim

This study aimed to assess HRQOL of Chinese patients with PNH, and identify key determinants, supply objective and factual data to policymakers and researchers and call on the whole society to pay attention to PNH and implement favorable policies about rare disease.

### Design and setting of the study

In 2022, a cross-sectional survey was conducted in PNH China, a legally recognized public welfare patient mutual aid organization established in April 2012. The organization comprised over 400 members, consisting of patients, their families, volunteers, and dedicated physicians and specialists. The survey assessed the patients’ HRQOL measured by 5L, socio-demographic, and clinical characteristics using a self-administered structured questionnaire through a professional online survey platform. The inclusion criteria were: (i) Accessible and willing to join in the study. (ii) Clinical diagnosis of PNH. Prior to the survey, investigators, who were managers of PNH China and well-trained PNH patients, provided a detailed introduction to the patients regarding the survey’s purpose, process, rights, and the questionnaire. After providing informed consent, patients could choose to either participate in the survey or directly opt out. If the patients were unable to complete the questionnaire, their guardians were authorized to do so on their behalf.

The study was approved by the Biomedical Ethics Committee of Dalian Medical University.

### EQ-5D-5L

5L contains a descriptive system including five dimensions: Mobility (MO), Self-Care (SC), Usual Activities (UA), Pain/Discomfort (PD), Anxiety/Depression (AD). Each dimension has five response levels: no problems, slight problems, moderate problems, severe problems, and extreme problems/unable to. The system thus defines a total of 3,125 (5^5^) health states. Each state can be translated into a single utility-based index score (i.e., health utility score) using a specific utility value set. Health utility score (HUS) is anchored on a scale from 0 (death) to 1 (full health), with a higher score indicating higher value of HRQOL [23–27]. In the study, the 5L HUS was calculated by adopting the 5L value set for China developed by Luo et al. [28] 5L also includes the EQ-VAS, recording the self-rated health status of the patients on a vertical visual analogue scale (VAS). The respondents rate their current health on a scale ranging from 0 (worst imaginable health status) to 100 (best imaginable health status) [25].

### Social-demographic and clinical characteristics

The questionnaire assessed the following social-demographic characteristics: gender, age, ethnicity, educational status, marital status, fertility situation, school/employment status, total personal income for the past year and health insurance. It also inquired about clinical characteristics, including initial symptoms of PNH, common complications during the past year (i.e., stones, thrombosis, renal failure, pulmonary hypertension, BMF, femoral head necrosis), misdiagnosis, and the latest pathological grade.

### Statistical analysis

The gathered data, exported in Excel format, was reviewed to eliminate any illogical patient data entries.

The social-demographic and clinical characteristics, and HRQOL of the patients were summarized using descriptive statistics with mean, standard deviations (SD), medians and interquartile ranges (IQR) for continuous variables; and frequencies, percentages for categorical variables.

Multiple linear regression analysis was utilized to explore the influence of above mentioned socio-demographic and clinical variables on HUS and VAS score (Table 1). To enhance the robustness of the analysis for the categorical variables, each category within a variable must have at least 16 observations according to expert opinion and statistical requirements. Variance inflation factor (VIF) was adopted to assess the multicollinearity among the independent variables, with the VIF value greater than 10 indicating the existence of multicollinearity. Multiple logistic regression was also used to assess the factors of self-reported EQ-5D problems. In the analysis, the responses to each of the five EQ-5D dimensions were classified as with and without problems. Five binary variables were thus generated and adopted as dependent variables in the five separate logistic models. An enter approach was adopted in the regression modelling, with the independent variables being coded as categorical variables and compared with a reference group.

**Table 1:** Assignment of independent variables

Variables	Variable definitions	Variable type
**Gender**	Male (0), Female (1)	Dichotomous variables
**Age**	Respondent’s age at the time of interview	Continuous quantitative variable
**Ethnicity**	Han (0), Other ethnicities (1)	Dichotomous variables
**Educational status**	Junior high school and below (1,0,0)	Multi-categorical variable
	High school, technical secondary school or vocational high school, etc. (0,1,0)	
	Junior college, college degree (0,0,0)	
	Postgraduate or above (0,0,1)	
**Marital status**	Have a partner (0), Single (1)	Dichotomous variables
**Fertility situation**	No (0), Yes (1)	Dichotomous variables
**School/employment status**	No (0), Yes (1)	Dichotomous variables
**Total personal income for the past year**	<35,128 CNY (0), ≥ 35,128 CNY (1)	Dichotomous variables
**Health insurance**	National Basic Medical Insurance [NO (0), Yes (1)], Commercial health insurance [NO (0), Yes (1)]	Dichotomous variables
**Initial symptom**	Hemoglobinuria [NO (0), Yes (1)]	Dichotomous variables
	Anemia (Fatigue, Tachycardia, Shortness of breath, Headache) [NO (0), Yes (1)]	
	Jaundice [NO (0), Yes (1)]	
	Pancytopenia, bone marrow hematopoietic failure [NO (0), Yes (1)]	
	Abdominal pain [NO (0), Yes (1)]	
	Back pain [NO (0), Yes (1)]	
	Erectile dysfunction [NO (0), Yes (1)]	
	Dysphagia [NO (0), Yes (1)]	
	Cognitive disorder [NO (0), Yes (1)]	
**Several common complications during the past year**	Stones [NO (0), Yes (1)]	Dichotomous variables
	Thrombosis [NO (0), Yes (1)]	
	Renal failure [NO (0), Yes (1)]	
	Pulmonary hypertension [NO (0), Yes (1)]	
	Bone marrow failure (BMF) [NO (0), Yes (1)]	
	Necrosis of the femoral head [NO (0), Yes (1)]	
**Misdiagnosis**	No (0), Yes (1)	Dichotomous variables
**The latest pathological grade**	Classic PNH (0,0)	Multi-categorical variable
	PNH in the setting of another specified bone marrow disorder (1,0)	
	Sub-clinical PNH (0,1)	

35,128: The 2021 annual average per capita disposable income of Chinese residents

CNY: Chinese Yuan

All statistical analyses were performed using SPSS (version 24). The statistical significance of this study was set at 0.05 level.

## Results

A total of 332 patients filled the questionnaire. Data from three patients with logical errors were excluded, and the other 329 patients from 26 provinces, autonomous regions and municipalities were included in the subsequent analysis.

### Social-demographic and clinical characteristics of PNH patients

**Fig. 1:**
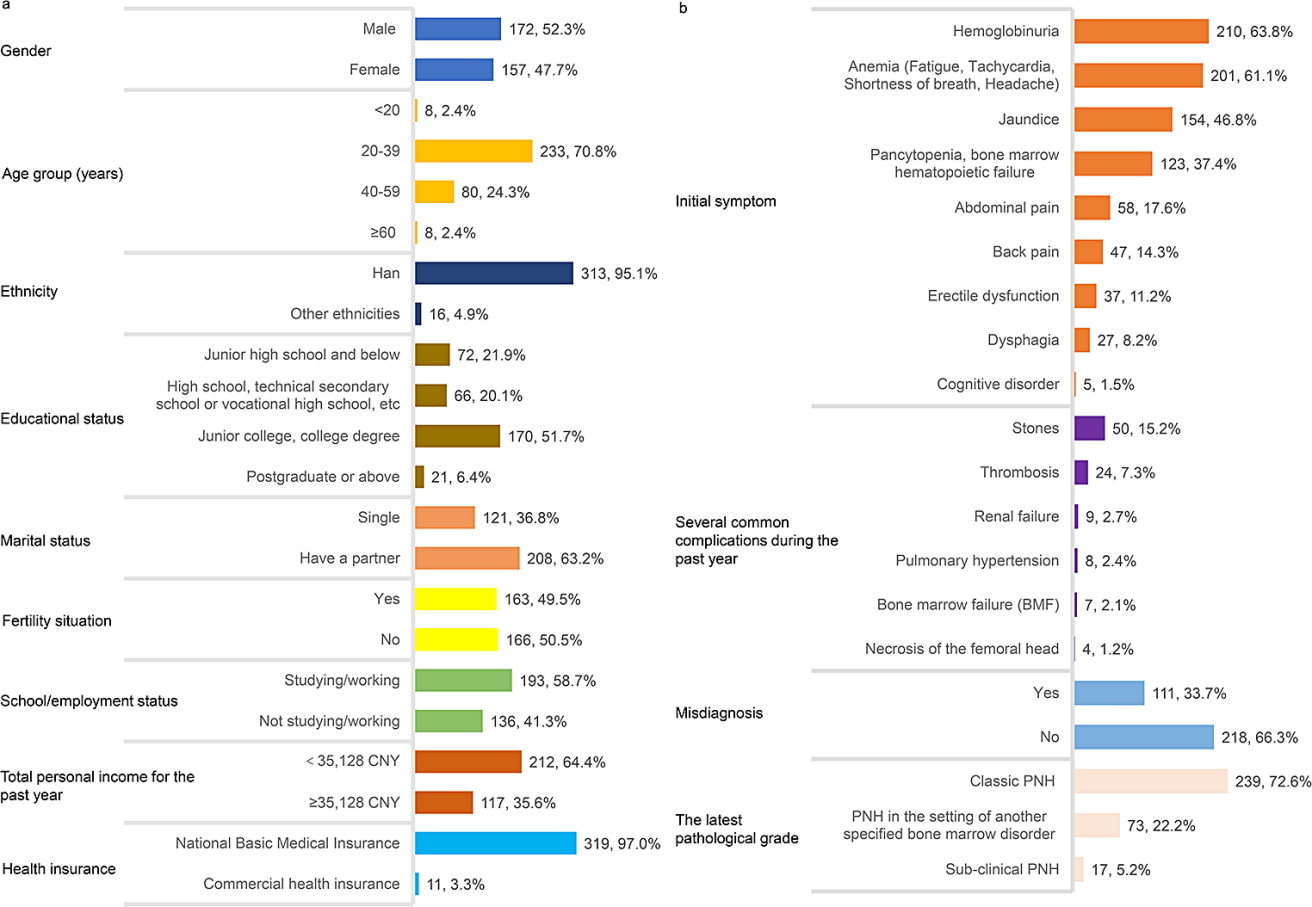
Indicators of social-demographic and clinical characteristics of all survey patients (N = 329). Age (years old): Mean = 35.3, SD = 10.0, Median = 34.0, IQR = 28.0–40.0, Range = 16.0–74.0. 35,128: the 2021 annual average per capita disposable income of Chinese residents. CNY: Chinese Yuan

The characteristics of the PNH patients are summarized in Fig. 1. Their mean (SD) age was 35.3 (10.0) years and median age (IQR) was 34.0 (28.0–40.0) years, with 52.3% of the patients being male. The majority of them (70.8%) were between 20 and 39 years, and 58.1% of the patients had attained an education level of junior college or above. A significant majority of patients (64.4%) reported an annual income that fell below the 2021 annual average per capita disposable income of Chinese residents (i.e., 35,128 CNY) [29].

Among the patients, 72.6% were diagnosed with classic PNH and 22.2% were diagnosed with PNH in the setting of another specified bone marrow disorder. A significant proportion of patients (63.8%) exhibited hemoglobinuria as an initial symptom. Anemia (fatigue, tachycardia, shortness of breath, headache) constituted the initial symptoms for 61.1% of the patients. Additionally, 15.2% of the patients experienced stone-related complications, and 7.3% presented with thrombosis during the past year. A misdiagnosis was reported by 33.7% of patients. (Fig. 1)

The median duration (IQR) from onset of symptoms to the time of the survey was 89.0 (44.0–155.5) months, and the mean duration (SD) was 104.7 (74.9) months. In the past year, 80.2% of 329 patients surveyed received treatment, whereas 19.8% did not. Among the patients with treatment, 91.3% were administered medication for symptomatic supportive care. Furthermore, 62.1% of these patients underwent red blood cell/platelet transfusion, and 18.2% were treated with novel complement inhibitors, all of which were provided as clinical donations. The proportion of low-dose combined chemotherapy was 1.1%; and hematopoietic stem cell transplant treatment was 0.4%.

### Health-related quality of life of PNH patients

**Fig. 2:**
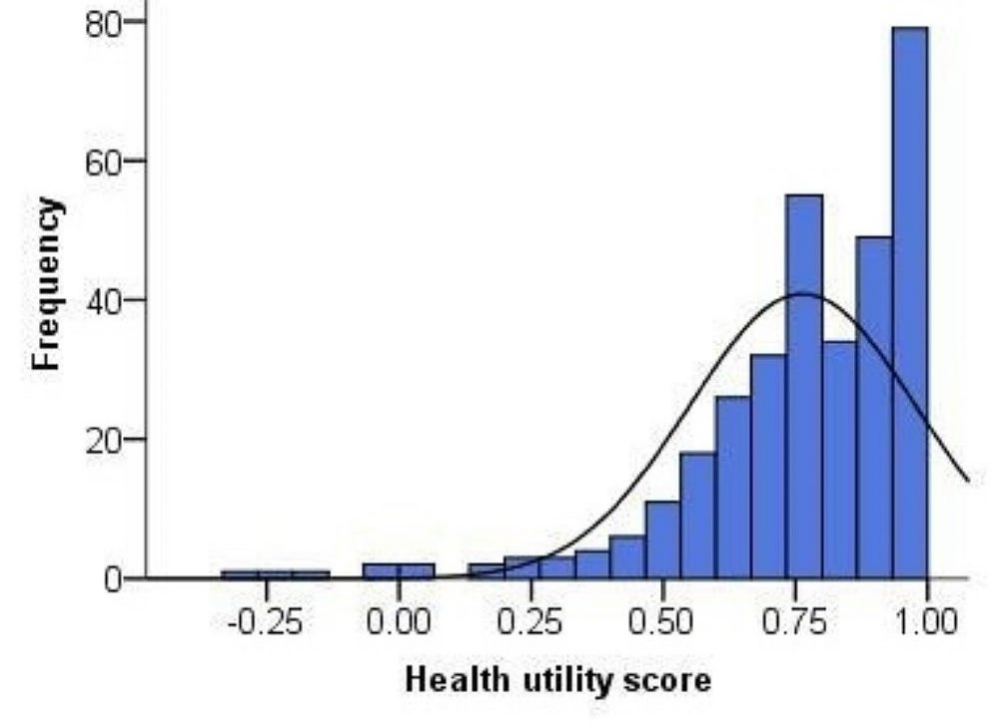
Frequency distribution of EQ-5D-5L health utility score. (Mean = 0.76, SD = 0.21, Median = 0.78, IQR = 0.68–0.91)

**Fig. 3:**
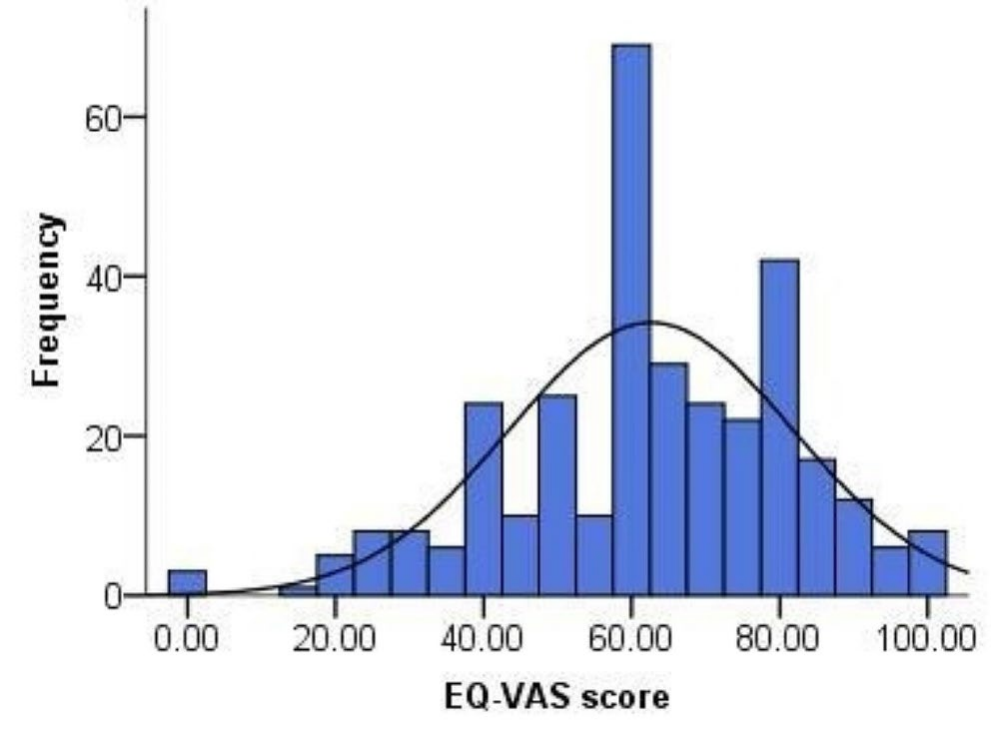
Frequency distribution of EQ-5D-5L-VAS score. (Mean = 62.61, SD = 19.20, Median = 62.00, IQR = 50.00–79.00)

The proportion of reporting problems in AD dimension (81.5%) was higher than the prevalence in the other dimensions (PD: 69.9%, UA: 53.5%, MO: 51.4%, SC: 13.4%) (Table 2), and thus only 9.1% of the patients reported full health defined by the 5L (i.e., no problems in all the 5 dimensions). The distribution of the 5L HUS was negatively skewed (Fig. 2), with the mean (SD) and median (IQR) values being 0.76 (0.21) and 0.78 (0.68–0.91). Similarly, the distribution of VAS score was also negatively skewed (Fig. 3), with the mean (SD) and median (IQR) values being 62.61 (19.20) and 62.00 (50.00–79.00), respectively.

**Table 2:** Sample distribution of EQ-5D-5L [N (%)] of Chinese PNH patients

Dimensions	Mobility	Self-care	Usual Activities	Pain/Discomfort	Anxiety/Depression
**No problems**	160 (48.6)	285 (86.6)	153 (46.5)	99 (30.1)	61 (18.5)
**Slight problems**	119 (36.2)	35 (10.6)	133 (40.4)	175 (53.2)	143 (43.5)
**Moderate problems**	38 (11.6)	4 (1.2)	34 (10.3)	41 (12.5)	82 (24.9)
**Severe problems**	9 (2.7)	5 (1.5)	7 (2.1)	10 (3.0)	36 (10.9)
**Extreme problems/Unable to**	3 (0.9)	0 (0.0)	2 (0.6)	4 (1.2)	7 (2.1)

Tables 3 and 4 present the 5L HUS and EQ-VAS score across various subgroups. Males had the mean (SD) HUS and VAS score of 0.79 (0.20) and 63.46 (19.47), which are higher than those of females: 0.73 (0.22) for HUS and 61.68 (18.91) for VAS. Compared to the patients older than 20 years, those younger patients reported the highest mean (SD) HUS of 0.80 (0.13) while having the lowest VAS score of 57.75 (20.50). Those with a higher socio-economic status (e.g., better educated, higher income and people who are studying or working) generally exhibited higher HUS and VAS score. The patients who showed initial symptoms and presented with common PNH symptoms had worse HRQOL than those who did not manifest these symptoms. For instance, the patients who exhibited hemoglobinuria as their initial symptom had lower mean (SD) HUS and VAS score of 0.75 (0.22) and 60.42 (19.63), respectively, compared to those who did not show signs of hemoglobinuria with the score being 0.78 (0.19) and 66.48 (17.83). Furthermore, compared to the patients without complications during the past year, those with complication(s) had lower HRQOL. For example, the patients without thrombosis had the mean HUS (SD) of 0.78 (0.20) and VAS score of 63.57 (18.38). In contrast, those with thrombosis reported the mean (SD) HUS of 0.59 (0.32) and VAS score of 50.42 (24.93). Among the patients with different kinds of pathological grades, the patients in the setting of another specified bone marrow disorder exhibited the lowest mean (SD) HUS and VAS score of 0.74 (0.21) and 58.81 (21.31); the patients with the subclinical type had the highest mean (SD) HUS and VAS score of 0.81 (0.23) and 72.59 (21.87).

**Table 3:** The EQ-5D-5L health utility score (HUS) and EQ-VAS score of various subgroups (social-demographic characteristics)

Variables	N	HUS		VAS score	
		Mean	SD	Mean	SD
**Gender**					
Male	172	0.79	0.20	63.46	19.47
Female	157	0.73	0.22	61.68	18.91
**Age group (years)**					
< 20	8	0.80	0.13	57.75	20.50
20–39	233	0.77	0.21	62.73	18.89
40–59	80	0.75	0.22	62.63	20.12
≥ 60	8	0.70	0.34	63.88	20.67
**Ethnicity**					
Han	313	0.76	0.22	62.72	19.09
Other ethnicities	16	0.80	0.14	60.50	21.76
**Educational status**					
Junior high school and below	72	0.70	0.31	56.07	20.44
High school, technical secondary school or vocational high school, etc.	66	0.76	0.19	60.48	19.54
Junior college, college degree	170	0.78	0.17	65.51	17.79
Postgraduate or above	21	0.82	0.14	68.24	19.26
**Marital status**					
Have a partner	208	0.76	0.21	63.07	19.31
Single	121	0.77	0.22	61.82	19.05
**Fertility situation**					
Yes	163	0.76	0.22	62.69	19.48
No	166	0.77	0.21	62.54	18.97
**School/employment status**					
Yes	193	0.80	0.19	65.46	18.63
No	136	0.71	0.24	58.57	19.34
**Total personal income for the past year**					
<35,128 CNY	212	0.73	0.24	59.29	19.29
≥ 35,128 CNY	117	0.82	0.14	68.63	17.56
**Health insurance**					
National Basic Medical Insurance					
Yes	319	0.76	0.21	62.22	19.17
No	10	0.77	0.25	75.00	16.60
Commercial health insurance					
Yes	11	0.86	0.12	69.09	27.55
No	318	0.76	0.22	62.39	18.86

HUS: Health utility score

VAS: Visual analogue scale

SD: Standard deviation

35,128: The 2021 annual average per capita disposable income of Chinese residents

CNY: Chinese Yuan

**Table 4:** The EQ-5D-5L health utility score (HUS) and EQ-VAS score of various subgroups (clinical characteristics)

Variables	N	HUS		VAS score	
		Mean	SD	Mean	SD
**Initial symptom**					
**Hemoglobinuria**					
Yes	210	0.75	0.22	60.42	19.63
No	119	0.78	0.19	66.48	17.83
**Anemia (Fatigue, Tachycardia, Shortness of breath, Headache)**					
Yes	201	0.73	0.23	60.30	18.95
No	128	0.82	0.18	66.23	19.09
**Jaundice**					
Yes	154	0.74	0.22	60.95	19.68
No	175	0.78	0.20	64.07	18.69
**Pancytopenia, bone marrow hematopoietic failure**					
Yes	123	0.73	0.24	61.38	20.32
No	206	0.78	0.19	63.34	18.50
**Abdominal pain**					
Yes	58	0.68	0.28	56.72	20.91
No	271	0.78	0.19	63.87	18.61
**Back pain**					
Yes	47	0.61	0.33	54.36	19.15
No	282	0.79	0.18	63.99	18.89
**Erectile dysfunction**					
Yes	37	0.76	0.24	63.92	17.94
No	292	0.76	0.21	62.45	19.37
**Dysphagia**					
Yes	27	0.66	0.29	56.37	15.91
No	302	0.77	0.20	63.17	19.39
**Cognitive disorder**					
Yes	5	0.81	0.13	53.40	33.31
No	324	0.76	0.22	62.75	18.95
**Several common complications during the past year**					
**Stones**					
Yes	50	0.71	0.21	60.02	22.24
No	279	0.77	0.21	63.08	18.61
**Thrombosis**					
Yes	24	0.59	0.32	50.42	24.93
No	305	0.78	0.20	63.57	18.38
**Renal failure**					
Yes	9	0.58	0.33	55.11	22.05
No	320	0.77	0.21	62.82	19.11
**Pulmonary hypertension**					
Yes	8	0.64	0.24	48.88	23.64
No	321	0.77	0.21	62.95	18.99
**Bone marrow failure (BMF)**					
Yes	7	0.44	0.28	40.14	10.88
No	322	0.77	0.21	63.10	19.05
**Necrosis of the femoral head**					
Yes	4	0.75	0.11	53.75	22.04
No	325	0.76	0.22	62.72	19.17
**Misdiagnosis**					
Yes	111	0.75	0.24	62.68	17.83
No	218	0.77	0.20	62.58	19.90
**The latest pathological grade**					
Classic PNH	239	0.77	0.21	63.06	18.07
PNH in the setting of another specified bone marrow disorder	73	0.74	0.21	58.81	21.31
Sub-clinical PNH	17	0.81	0.23	72.59	21.87
**Total**	329	0.76	0.21	62.61	19.20

HUS: Health utility score

VAS: Visual analogue scale

SD: Standard deviation

### Influencing factors of EQ-5D-5L health utility score and EQ-VAS scores

Negative correlation with HUS was observed with three clinical factors including initial symptoms of anemia (fatigue, tachycardia, shortness of breath, headache) (B=-0.057, 95%CI: [-0.105,-0.010], p = 0.017), back pain (B=-0.146, 95%CI: [-0.214,-0.078], p = 0.000), and complication of thrombosis (B=-0.161, 95%CI: [-0.245,-0.077], p = 0.000) (Table 5).

**Table 5:** Factors associated with EQ-5D-5L health utility score of Chinese PNH patients

Independent variable	Coefficient (95%CI)	P	VIF
**Constant**	0.908 (0.781,1.035)	0.000	
**Gender (Male)**			
Female	-0.044(-0.091,0.003)	0.067	1.258
**Age**	-0.002(-0.004,0.001)	0.228	1.604
**Ethnicity (Han)**			
Other ethnicities	-0.001(-0.101,0.100)	0.988	1.079
**Educational status (Junior college, college degree)**			
Junior high school and below	-0.031 (-0.091,0.029)	0.315	1.420
High school, technical secondary school or vocational high school, etc.	-0.011 (-0.071,0.049)	0.720	1.343
Postgraduate or above	0.010 (-0.080,0.101)	0.823	1.129
**Marital status (Have a partner)**			
Single	0.011 (-0.046,0.067)	0.716	1.727
**Fertility situation (No)**			
Yes	0.013 (-0.046,0.073)	0.654	2.005
**School/employment status (No)**			
Yes	0.046 (-0.006,0.099)	0.083	1.534
**Total personal income for the past year (<35,128 CNY)**			
≥ 35,128 CNY	0.048 (-0.009,0.104)	0.096	1.657
**Hemoglobinuria (No)**			
Yes	-0.015 (-0.063,0.033)	0.527	1.219
**Anemia (Fatigue, Tachycardia, Shortness of breath, Headache) (No)**			
Yes	-0.057 (-0.105,-0.010)	**0.017**	1.208
**Jaundice (No)**			
Yes	0.006 (-0.042,0.055)	0.804	1.346
**Pancytopenia, bone marrow hematopoietic failure (No)**			
Yes	-0.030 (-0.076,0.016)	0.199	1.133
**Abdominal pain (No)**			
Yes	-0.003 (-0.065,0.060)	0.935	1.301
**Back pain (No)**			
Yes	-0.146 (-0.214,-0.078)	**0.000**	1.317
**Erectile dysfunction (No)**			
Yes	0.029 (-0.047,0.105)	0.456	1.331
**Dysphagia (No)**			
Yes	-0.052 (-0.137,0.032)	0.226	1.236
**Stones (No)**			
Yes	-0.040 (-0.101,0.021)	0.198	1.102
**Thrombosis (No)**			
Yes	-0.161 (-0.245,-0.077)	**0.000**	1.101
**Misdiagnosis (No)**			
Yes	-0.036 (-0.083,0.010)	0.122	1.097
**The latest pathological grade (Classic PNH)**			
PNH in the setting of another specified bone marrow disorder	-0.028 (-0.082,0.026)	0.307	1.154
Sub-clinical PNH	-0.009 (-0.109,0.091)	0.862	1.129
**Adjusted R** ^**2**^	0.193		

CI: Confidence interval

VIF: Variance inflation factor

P: p value

35,128: The 2021 annual average per capita disposable income of Chinese residents

CNY: Chinese Yuan

The analysis of VAS score revealed that the patients with the income more than 35,128 CNY in the past year exhibited higher score (B = 5.708, 95%CI: [0.464,10.951], p = 0.033). VAS score was negatively predicted by the initial symptom of hemoglobinuria (B=-5.226, 95%CI: [-9.708,-0.745], p = 0.022) and presence of thrombosis (B=-10.330, 95%CI: [-18.198,-2.463], p = 0.010) (Table 6).

**Table 6:** Factors associated with EQ-VAS score of Chinese PNH patients

Independent variable	Coefficient (95%CI)	P	VIF
**Constant**	70.136 (58.285,81.987)	0.000	
**Gender (Male)**			
Female	-0.153 (-4.532,4.226)	0.945	1.258
**Age**	0.025 (-0.223,0.273)	0.843	1.604
**Ethnicity (Han)**			
Other ethnicities	-5.460 (-14.875,3.956)	0.255	1.079
**Educational status (Junior college, college degree)**			
Junior high school and below	-5.356 (-10.975,0.263)	0.062	1.420
High school, technical secondary school or vocational high school, etc.	-4.038 (-9.681,1.605)	0.160	1.343
Postgraduate or above	1.660 (-6.814,10.135)	0.700	1.129
**Marital status (Have a partner)**			
Single	0.354 (-4.960,5.667)	0.896	1.727
**Fertility situation (No)**			
Yes	-0.190 (-5.712,5.333)	0.946	2.005
**School/employment status (No)**			
Yes	1.961 (-2.944,6.866)	0.432	1.534
**Total personal income for the past year (<35,128 CNY)**			
≥ 35,128 CNY	5.708 (0.464,10.951)	**0.033**	1.657
**Hemoglobinuria (No)**			
Yes	-5.226 (-9.708,-0.745)	**0.022**	1.219
**Anemia (Fatigue, Tachycardia, Shortness of breath, Headache) (No)**			
Yes	-4.248 (-8.643,0.147)	0.058	1.208
**Jaundice (No)**			
Yes	-0.285 (-4.817,4.248)	0.902	1.346
**Pancytopenia, bone marrow hematopoietic failure (No)**			
Yes	-0.844 (-5.134,3.446)	0.699	1.133
**Abdominal pain (No)**			
Yes	-1.494 (-7.329,4.342)	0.615	1.301
**Back pain (No)**			
Yes	-6.092 (-12.487,0.303)	0.062	1.317
**Erectile dysfunction (No)**			
Yes	6.600 (-0.520,13.719)	0.069	1.331
**Dysphagia (No)**			
Yes	-3.645 (-11.542,4.251)	0.364	1.236
**Stones (No)**			
Yes	-2.579 (-8.281,3.123)	0.374	1.102
**Thrombosis (No)**			
Yes	-10.330 (-18.198,-2.463)	**0.010**	1.101
**Misdiagnosis (No)**			
Yes	-1.491 (-5.811,2.830)	0.498	1.097
**The latest pathological grade (Classic PNH)**			
PNH in the setting of another specified bone marrow disorder	-4.300 (-9.341,0.740)	0.094	1.154
Sub-clinical PNH	5.157 (-4.201,14.515)	0.279	1.129
**Adjusted R** ^**2**^	0.123		

CI: Confidence interval

VIF: Variance inflation factor

P: p value

35,128: The 2021 annual average per capita disposable income of Chinese residents

CNY: Chinese Yuan

Variance inflation factor (VIF) value of all variables in the two linear models was less than 10, indicating the absence of multicollinearity.

### Influencing factors of EQ-5D-5L problems

Significant factors influencing the 5L problems were shown in Table 7. Patients with initial symptoms of anemia (fatigue, tachycardia, shortness of breath, headache) experienced an increased risk of reporting problems in MO (OR [95%CI]: 2.37[1.40,4.02], p = 0.001), SC (OR [95%CI]: 3.69[1.46,9.38], p = 0.006), and UA (OR [95%CI]: 3.06[1.78,5.26], p = 0.000) dimensions. Compared to the patients with an income below 35,128 CNY, those who earned higher had experienced fewer SC problems (OR [95%CI]: 0.32[0.11,0.94], p = 0.038). Compared to male, female faced a higher risk of UA problems (OR [95%CI]: 1.79[1.04,3.08], p = 0.036). Moreover, compared with the patients who were neither studying nor employed, the patients who were studying or employed experienced less UA problems (OR [95%CI]: 0.51[0.28,0.94], p = 0.032). Compared with the patients without complications of thrombosis and stones during the past year, the patients with complications of stones (OR [95%CI]: 2.78[1.15,6.71], p = 0.023) and thrombosis (OR [95%CI]: 5.30[1.15,24.55], p = 0.033) experienced an increased risk of PD problems. The patients with sub-clinical PNH tended to report fewer AD problems than the patients with classic PNH (OR [95%CI]: 0.23[0.07,0.73], p = 0.013).

**Table 7:** Multivariate analyses evaluating associations of factors with each of the five health dimensions

Dimensions	Mobility		Self-Care		Usual Activities		Pain/Discomfort		Anxiety/Depression	
	OR (95%CI)	P	OR (95%CI)	P	OR (95%CI)	P	OR (95%CI)	P	OR (95%CI)	P
**Gender (Male)**										
Female	1.67(0.99,2.82)	0.055	0.96(0.44,2.10)	0.915	1.79(1.04,3.08)	**0.036**	1.13(0.64,2.01)	0.666	1.67(0.85,3.27)	0.136
**Age**	1.03(0.99,1.06)	0.118	1.03(0.98,1.07)	0.230	1.02(0.99,1.05)	0.236	1.02(0.98,1.05)	0.395	1.01(0.97,1.04)	0.806
**Ethnicity (Han)**										
Other ethnicities	1.41(0.46,4.32)	0.544	0.52(0.06,4.72)	0.565	1.96(0.59,6.54)	0.274	1.44(0.41,5.01)	0.567	0.96(0.24,3.90)	0.959
**Educational status (Junior college, college degree)**										
Junior high school and below	0.63(0.32,1.23)	0.172	1.19(0.49,2.93)	0.700	0.54(0.27,1.08)	0.081	0.80(0.38,1.68)	0.559	0.48(0.20,1.17)	0.107
High school, technical secondary school or vocational high school, etc.	1.37(0.69,2.70)	0.368	0.80(0.29,2.19)	0.662	1.55(0.76,3.16)	0.232	1.63(0.77,3.45)	0.206	0.44(0.19,1.01)	0.053
Postgraduate or above	0.91(0.34,2.46)	0.847	1.62(0.30,8.64)	0.574	1.66(0.59,4.64)	0.335	0.65(0.23,1.82)	0.410	2.30(0.48,11.03)	0.297
**Marital status (Have a partner)**										
Single	0.63(0.33,1.19)	0.156	0.93(0.36,2.41)	0.887	0.58(0.30,1.13)	0.107	0.62(0.32,1.23)	0.173	2.07(0.89,4.82)	0.090
**Fertility situation (No)**										
Yes	0.96(0.49,1.86)	0.895	1.33(0.49,3.63)	0.582	0.82(0.41,1.63)	0.570	0.82(0.40,1.70)	0.602	1.23(0.54,2.80)	0.621
**School/employment status (No)**										
Yes	0.58(0.32,1.05)	0.071	0.58(0.25,1.34)	0.202	0.51(0.28,0.94)	**0.032**	0.68(0.36,1.29)	0.239	0.61(0.28,1.31)	0.200
**Total personal income for the past year (<35,128 CNY)**										
≥ 35,128 CNY	0.74(0.39,1.38)	0.339	0.32(0.11,0.94)	**0.038**	0.85(0.44,1.64)	0.630	1.07(0.55,2.08)	0.846	0.77(0.35,1.68)	0.508
**Hemoglobinuria (No)**										
Yes	1.04(0.61,1.77)	0.886	0.93(0.42,2.05)	0.858	0.89(0.51,1.54)	0.671	0.77(0.43,1.39)	0.387	0.82(0.41,1.66)	0.587
**Anemia (Fatigue, Tachycardia, Shortness of breath, Headache) (No)**										
Yes	2.37(1.40,4.02)	**0.001**	3.69(1.46,9.38)	**0.006**	3.06(1.78,5.26)	**0.000**	1.68(0.97,2.92)	0.065	1.22(0.64,2.34)	0.546
**Jaundice (No)**										
Yes	0.98(0.57,1.68)	0.932	0.62(0.27,1.46)	0.275	1.68(0.96,2.93)	0.069	1.15(0.64,2.05)	0.646	1.24(0.63,2.48)	0.534
**Pancytopenia, bone marrow hematopoietic failure (No)**										
Yes	1.45(0.87,2.42)	0.156	1.35(0.65,2.81)	0.422	1.23(0.72,2.08)	0.455	1.16(0.65,2.05)	0.620	0.80(0.41,1.56)	0.518
**Abdominal pain (No)**										
Yes	0.59(0.30,1.18)	0.134	0.45(0.15,1.33)	0.150	0.58(0.29,1.20)	0.143	0.93(0.41,2.08)	0.853	1.44(0.51,4.05)	0.494
**Back pain (No)**										
Yes	1.41(0.66,3.03)	0.375	2.57(0.94,7.03)	0.066	2.15(0.95,4.84)	0.066	2.02(0.77,5.31)	0.153	2.06(0.62,6.83)	0.236
**Erectile dysfunction (No)**										
Yes	0.93(0.41,2.13)	0.870	0.92(0.28,3.02)	0.884	0.60(0.26,1.40)	0.234	0.78(0.30,1.99)	0.601	1.13(0.39,3.30)	0.824
**Dysphagia (No)**										
Yes	1.73(0.67,4.44)	0.257	2.02(0.56,7.19)	0.281	1.01(0.38,2.66)	0.982	3.43(0.90,13.03)	0.070	1.08(0.29,4.02)	0.914
**Stones (No)**										
Yes	1.40(0.70,2.81)	0.344	0.92(0.35,2.46)	0.872	2.03(0.98,4.22)	0.058	2.78(1.15,6.71)	**0.023**	1.75(0.64,4.75)	0.273
**Thrombosis (No)**										
Yes	2.08(0.80,5.40)	0.133	2.22(0.66,7.55)	0.200	1.56(0.60,4.08)	0.363	5.30(1.15,24.55)	**0.033**	4.03(0.51,31.98)	0.187
**Misdiagnosis (No)**										
Yes	1.33(0.79,2.23)	0.286	1.89(0.90,3.97)	0.093	1.05(0.62,1.79)	0.851	1.21(0.68,2.14)	0.516	1.14(0.58,2.24)	0.697
**The latest pathological grade (Classic PNH)**										
PNH in the setting of another specified bone marrow disorder	1.33(0.73,2.43)	0.357	1.20(0.50,2.84)	0.683	1.86(0.99,3.50)	0.055	1.57(0.80,3.06)	0.190	0.92(0.43,1.94)	0.822
Sub-clinical PNH	1.41(0.47,4.26)	0.540	2.77(0.62,12.32)	0.182	0.63(0.19,2.10)	0.456	0.33(0.10,1.07)	0.066	0.23(0.07,0.73)	**0.013**

CI: Confidence interval

OR: Odds Ratio

P: p value

35,128: The 2021 annual average per capita disposable income of Chinese residents

CNY: Chinese Yuan

## Discussion

The study expounded the socio-demographic and clinical characteristics of Chinese PNH patients and assessed their HRQOL based on a sample with better representativeness. Furthermore, we explored the determinants influencing HRQOL of the patients within the Chinese context. Hence, the study has deepened the understanding of the profile and HRQOL of PNH patients in China.

Consistent with previous findings [8, 13, 30–32], 95.1% of the patients fell between 20 and 59 years, and 70.8% were even between 20 and 39 years in our study. These suggest that PNH predominantly manifests in early adulthood and could affect individuals during their most productive years. A worrying 64.4% of the patients reported annual income below the 2021 annual average per capita disposable income of Chinese residents, implying potential economic strain or burden due to the disease [33–35]. Hence, to reduce its impact on productivity, the efficient control of the disease, particularly the management of hemolysis (the major characteristic of PNH [36–39]), is required.

Of significant note, our study revealed that 63.8% of the patients initially presented with hemoglobinuria, a clinical hallmark of PNH [8, 18]. This observation has interesting resonance with the report by Lee et al. [40] that hemoglobinuria was a significant predictor of thromboembolism in Asian patients with PNH. Similarly, other studies [36, 41] have also shown that PNH-related symptoms, such as hemoglobinuria or dyspnea, increase the risk of thrombosis. Those reinforce the exigency of research to delineate the relationship between clinical manifestations of PNH and thrombosis susceptibility, thereby facilitating the evolution of efficacious prophylactic and therapeutic approaches.

Consistent with previous evidence [14, 42–44], the misdiagnosis rate was as high as 33.7% in our study. This means that the accurate diagnosis of PNH remains challenging, though its symptoms are common [30, 36, 45–49]. The high rate may have serious implications including prolonged pain, exacerbating mental health challenges and rising medical costs [35]. Therefore, we urgently need to refine existing PNH diagnosis and treatment strategies to raise clinician awareness and enhance the timely and accurate diagnosis.

Compared to HRQOL of the general Chinese population measured by 5L [50], our sample demonstrated a markedly inferior HRQOL. Specifically, the proportions of reporting problems in four dimensions (AD: 81.5% vs. 47.2%, PD: 69.9% vs. 44.8%, UA: 53.5% vs. 25.1%, and MO: 51.4% vs. 26.8%) were much higher which could be attributed to the fear of PNH leading to a feeling of “life limitation” and “frailty” in these patients [51]. The average 5L HUS and VAS score (i.e., 0.76 & 62.61) also trailed significantly behind those of the general Chinese population who achieved mean of 0.93 and 84.57. Such divergences underscore the unique challenges faced by PNH patients in China, particularly in the AD dimension, highlighting the considerable psychological burden associated with PNH. Hence, a holistic healthcare approach integrating both mental and physical aspects is imperative for PNH patients in China. It is noteworthy that the proportion of SC problems was lower in our study (13.4% vs. 23.7%). This may be due to their high level of concern for their own health, regular medical follow-up, and the need for disease management. Moreover, the mean age of the general Chinese population was older (42.8 years vs. 35.3 years), and older individuals are more frequently encounter self-care challenges.

The regression results showed that demographic characteristics, gender, school/employment status, total personal income, and clinical characteristics such as initial symptoms of hemoglobinuria, anemia (fatigue, tachycardia, shortness of breath, headache), and back pain, complications of stones, thrombosis, and pathological grade were significant factors exerting differential effects on HUS, VAS score, or certain EQ-5D dimensions in Chinese PNH patients.

Gender was the key factor associated with UA problems (OR = 1.79), which may be attributed to multiple factors including cultural and social expectations. Meanwhile, the patients with PNH at school or in the work environment were at lower risk of UA problems relative to patients who were not at school or employed (OR = 0.51). This may be because these settings provide better support and adaptation, as well as opportunities to promote patients’ participation in daily activities and to maintain function [52–54]. High-income patients were less likely to face SC problems (OR: 0.32) and performed better on VAS score (p < 0.05), which may be because they could afford high-quality medical services. The finding is consistent with earlier studies and further confirms the strong association between socioeconomic factors and health status [53–57].

Our findings confirm that the initial symptoms of PNH (i.e., hemoglobinuria, anemia [fatigue, tachycardia, shortness of breath, headache] and back pain) have a significantly negative impact on HRQOL of the patients. The symptoms not only lead to physical discomfort, but also aggravate psychological burden, thus affecting the patient’s overall HRQOL [8, 34, 35, 51, 58, 59]. The logistic analysis further revealed that the patients with the symptoms performed significantly worse on several HRQOL dimensions (MO, SC, UA) than the patients without the symptoms. These results thus highlight the importance of early identification, diagnosis and treatment of the patients with PNH to relieve the impact of the symptoms.

Thrombosis was a key factor affecting HRQOL, specifically on the PD dimension in our study. The finding is consistent with relevant findings from the international PNH registry [13] and further validates the clinical importance of preventing thrombosis in PNH management. Also, the patients with stones faced more problems on the PD dimension (OR = 2.78). These problems may not only limit a patient’s daily activities, but may also lead to mood swings and reduced sleep quality, thereby exacerbating anxiety and depressive symptoms [5, 35, 59–62]. Notably, patients with classic PNH were at higher risk of AD problems compared with those with sub-clinical PNH, suggesting that they may require more medical and psychological support.

Our research has identified key factors that influence specific dimensions of HRQOL in Chinese patients with PNH, such as physical functioning and mental health. Addressing these specific areas requires targeted and comprehensive measures. Firstly, existing health policies need to place greater emphasis on the recognition and management of early symptoms to mitigate the long-term impact of PNH. This can be achieved by integrating advanced screening technologies in primary care, increasing public health education, and developing more precise diagnostic and treatment guidelines. Secondly, the health system needs to integrate more effective disease management strategies, including patient education, psychological support, and the optimal allocation of medical resources. Furthermore, enhancing healthcare providers’ ability to recognize early symptoms of PNH is crucial. We recommend regular professional training and workshops to improve medical personnel’s sensitivity to PNH diagnoses. Collaboration with hematologists is also suggested to promote shared learning experiences and ensure a multidisciplinary approach to PNH management. Strengthening public awareness of PNH is also necessary. Media campaigns, community educational activities, and cooperation with patient support groups can effectively increase societal attention to this disease. Through these measures, we hope to improve the HRQOL for patients with PNH.

Our study has several limitations. First, the PNH patients recruited were all from a single institution, though it is the largest one for the patients in China. Second, we can not infer clear causality between HRQOL and the factors assessed due to the cross-sectional design. Although the associations provide useful initial insights into HRQOL in Chinese patients with PNH, an in-depth exploration of its longitudinal impact is still warranted. Third, the current sample size may have limited the detailed analysis of all variables.

## Conclusion

In summary, the study described the profile and HRQOL of PNH patients in China, and elucidated the significant socio-demographic and clinical characteristics affecting their HRQOL. The findings could be adopted as the evidence for enhancing health status of PNH patients in China.

### Electronic Supplementary Material

Below is the link to the electronic supplementary material.

Supplementary material 1: Fig. 1

## Data Availability

The data that support the findings of this study are available from PNH China, but restrictions apply to the availability of these data, which were used under licence for the current study and so are not publicly available. The data are, however, available from the authors upon reasonable request and with the permission of PNH China.

## Abbreviations

**PNH**: Paroxysmal nocturnal hemoglobinuria
**PIGA**: Phosphatidylinositol glycan class A
**TEs**: Thrombotic events
**BMF**: Bone marrow failure
**HRQOL**: Health-related quality of life
**5L**: EQ-5D-5L
**CNY**: Chinese Yuan
**MO**: Mobility
**SC**: Self-Care
**UA**: Usual Activities
**PD**: Pain/Discomfort
**AD**: Anxiety/Depression
**HUS**: Health utility score
**VAS**: Visual analogue scale
**SD**: Standard deviations
**IQR**: Interquartile ranges
**VIF**: Variance inflation factor
**CI**: Confidence interval
**P**: p value
**OR**: Odds Ratio
