# Supplementary figures and images for: Health-related quality of life and influencing factors of patients with paroxysmal nocturnal hemoglobinuria in China
Source: Orphanet J Rare Dis. 2024 May 3;19:186. doi: 10.1186/s13023-024-03178-x (PMC11067208; doi:10.1186/s13023-024-03178-x)

a

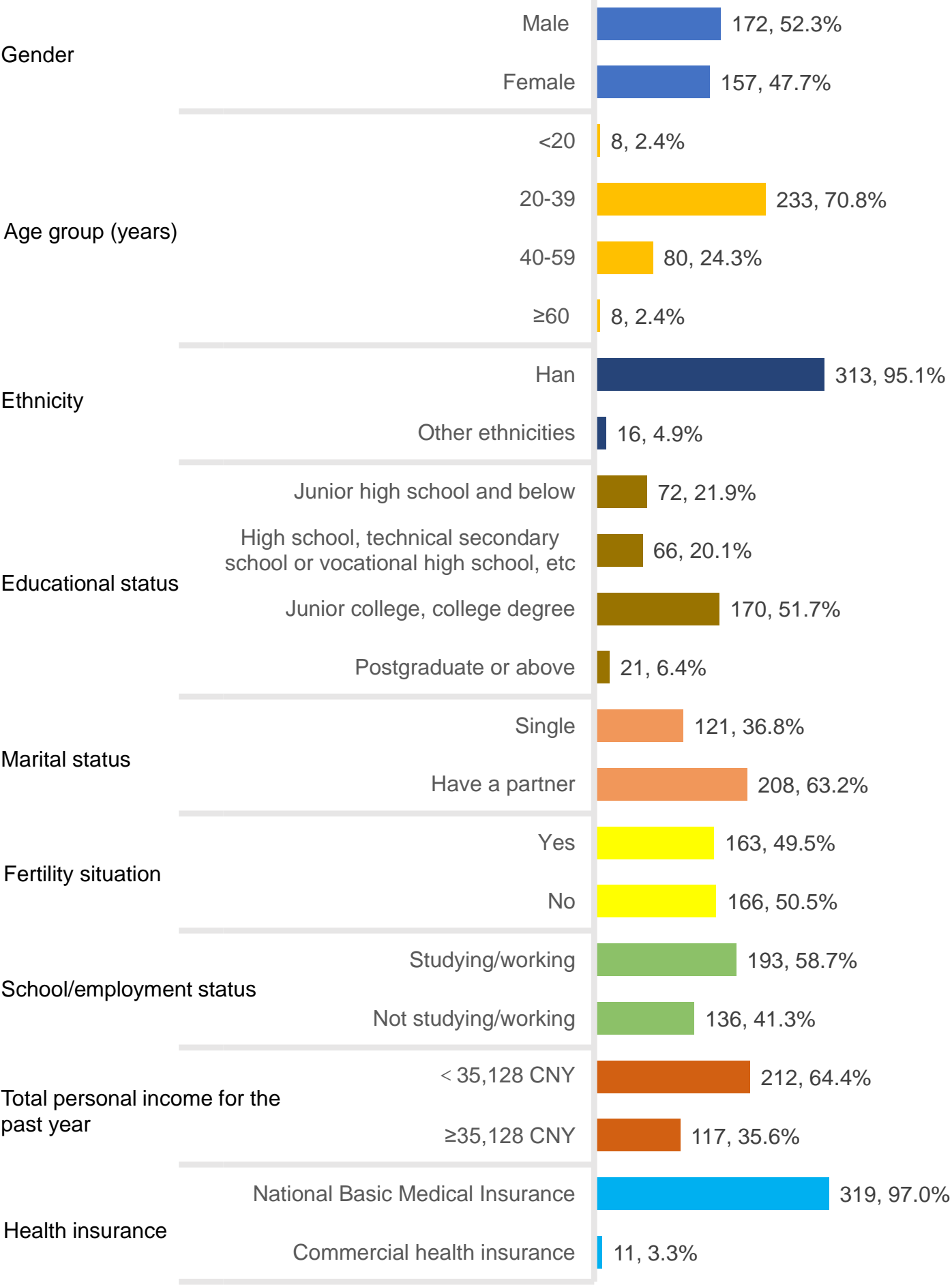

b

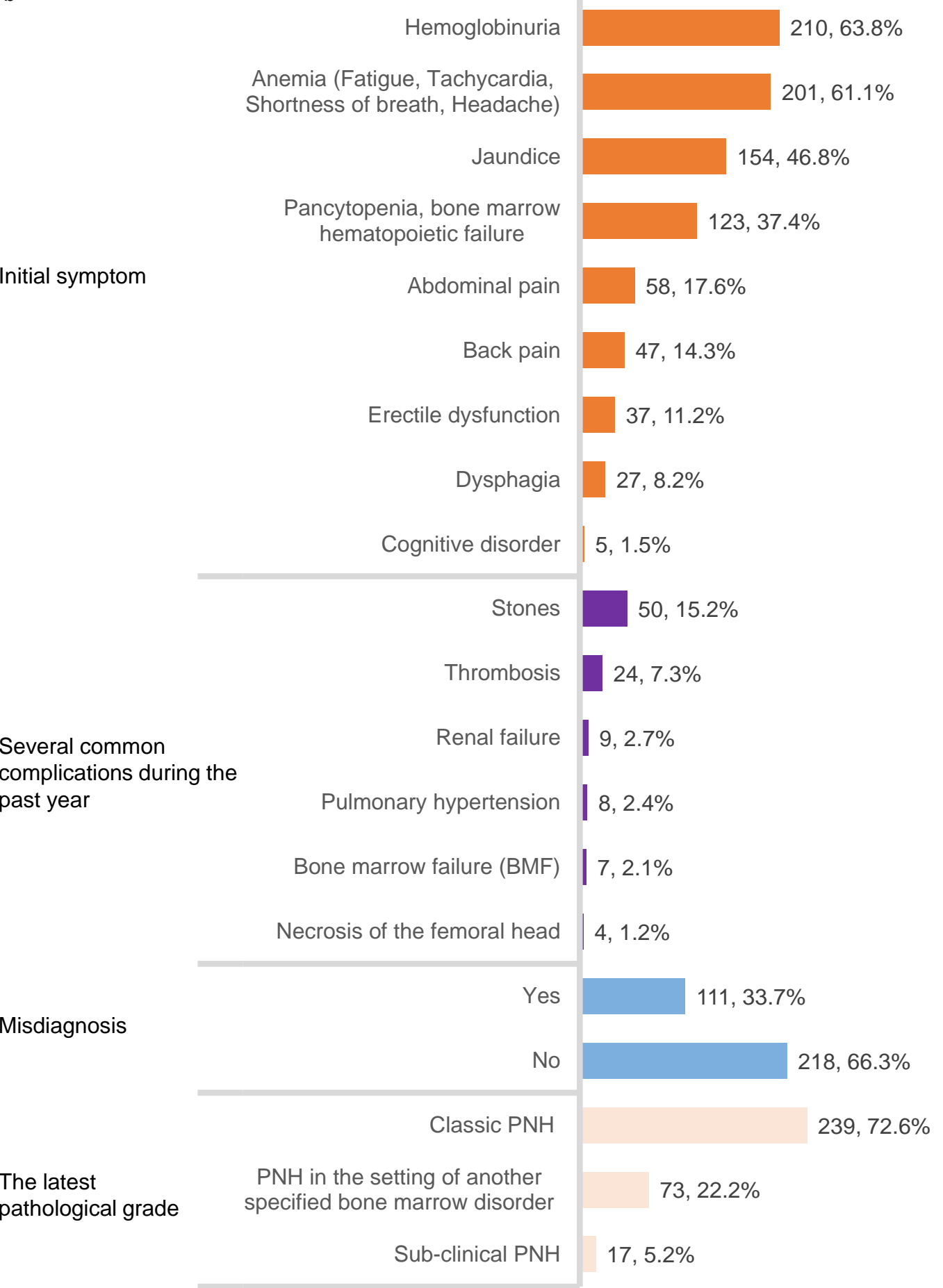

Supplement: Supplementary file 1 — Supplementary material 1: Fig. 1 [file 13023_2024_3178_MOESM1_ESM.pdf]
